# A Broken Heart: Cardiac Arrest As the Initial Presentation of Myasthenic Crisis

**DOI:** 10.7759/cureus.6891

**Published:** 2020-02-05

**Authors:** Alexander Andreev, Farhana Begum, Anjuli Singh, Sonu Sahni

**Affiliations:** 1 Internal Medicine, Brookdale University Hospital Medical Center, New York, USA; 2 Internal Medicine, New York Medical College, Valhalla, USA

**Keywords:** cardiac arrest, short pr interval, myasthenia gravis, conduction abnormalities, myasthenic crisis

## Abstract

Myasthenic crisis is a life-threatening condition commonly associated with respiratory failure and may present in unusual ways. However, there is paucity in the literature about the cardiac manifestations of myasthenia gravis. We present a case of a 61-year-old male who presented to the emergency room with upper respiratory infection symptoms who soon thereafter suffered sudden cardiac arrest. He was found to have shortened PR interval pre and post arrest onelectrocardiogram (EKG). Only past medical history, discovered post cardiac arrest, was myastenia gravis. All other causes of cardiac arrest were ruled out, and it was deemed to be due to a manifestation of myastenia gravis. The patient was treated with intravenous steroids and plasmapheresis with resolution of shortened PR interval. It is hypothesized that striatial muscle antibodies may trigger inflammation in cardiac muscle and cause conduction abnormalities. In addition, anti-Kv1.4 antibodies have been associated with EKG abnormalities, including QT prolongation and T-wave inversion. To our knowledge, we are the first to report myasthenic crisis manifesting with isolated cardiac arrest with pulseless electrical activity and a shortened PR interval.

## Introduction

Myasthenic crisis (MC) is a life-threatening condition commonly associated with respiratory failure [1]. MC is typically preceded by worsening of other myasthenia gravis (MG) symptoms, including limb, ocular and bulbar muscle weakness. However, there exists a paucity in the literature about cardiac manifestations of MG. There are isolated reports of possible arrhythmias and chronic heart disease associated with MG [2]. Herein we present, to the best of our knowledge, the first case to report MC manifesting with isolated cardiac arrest with pulseless electrical activity (PEA) and a shortened PR interval as the initial presentation of MC. An abstract version of this case was presented at the CHEST Annual Meeting October 19-23, 2019 in New Orleans, LA (Andreev A, Singh A, Begum F, Sahni S. Wednesday Medical Student/Resident Case Report Posters, https://journal.chestnet.org/article/S0012-3692(19)33523-8/fulltext). 

## Case presentation

**Figure 1:**
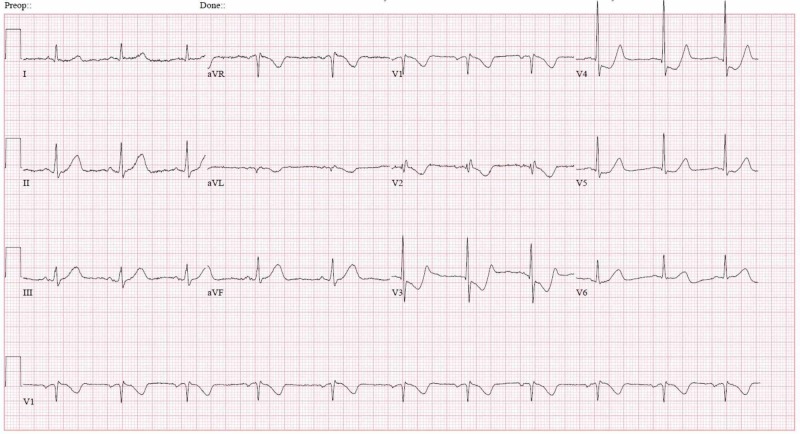
EKG showing normal sinus rhythm with short PR interval (96 ms) (post cardiac arrest)

A 61-year-old male presented to the emergency room with a three-day history of upper respiratory infection symptoms including cough, fever and myalgia. At presentation, he did not endorse any limb, ocular or bulbar weakness. Initial physical exam was unremarkable, and vital signs were within normal limits. Neurologic exam was also noted to be normal with no focal muscle weakness. Electrocardiogram (EKG) showed normal sinus rhythm with shortened PR interval of 96 ms (normal: 120-200ms). Shortly after the presentation, the patient was found to be unresponsive and pulseless. Telemetry monitoring was indicative of cardiac arrest with PEA. Advanced cardiac life support was initiated, the patient was subsequently intubated and cardiopulmonary resuscitation was continued. Return of spontaneous circulation (ROSC) occurred within seven minutes, and the patient was admitted to the medical intensive care unit (MICU). Post resuscitation EKG showed sinus tachycardia with shortened PR interval, similar to prior. The EKG immediately post cardiac arrest is shown in Figure 1.

Laboratory data on presentation were not obtained due to cardiac arrest, and first available set of laboratory investigations was after ROSC. Only significant abnormality noted on initial laboratory findings was lactic acidosis, which is expected after cardiac arrest. Lactate level quickly normalized within four hours' time. Laboratory results are shown in Table 1. Pulmonary embolism (PE) and pneumonia were highly suspected. However, CT angiogram of the chest was negative for PE and bronchoscopy was essentially normal. In addition, sepsis workup including blood cultures, chest x-ray and urinalysis were all found to be negative. Pre-excitation syndrome was ruled out by electrophysiology, and it was suggested that the shortened PR could be due to enhanced atrioventricular (AV) node conduction. A diagnosis remained unclear for the first three days of the MICU stay until the patient’s prior history of MG was verified by his primary care provider. Treatment with plasmapheresis and methylprednisolone was initiated resulting in significant improvement of the patient’s condition. Short PR-interval resolved after the completion of five sessions of plasmapheresis, and the patient was extubated shortly thereafter. The patient fully recovered and was discharged to regular follow-up and has remained stable thus far.

**Table 1:** Laboratory values (post cardiac arrest) ALT, alanine aminotransferase; AST, aspartate aminotransferase; Hgb, hemoglobin; INR, international normalized ratio; PLT, platelet; pCO_2_, partial pressure of carbon dioxide; pO_2_, partial pressure of oxygen; PT, prothrombin time; PTT, partial thromboplastin time; WBC, white blood cell

Laboratory Test (Normal Range)	Value
Hgb (12.9-16.7 g/dL)	10.6 g/dL
WBC (4.10-10.10 10x3/uL)	14.90 10x3/uL
PLT (153-338 10x3/uL)	318 10x3/uL
PT/INR	1.28
PTT (23.5-35.5 seconds)	29.5 sec
Glucose (70-99 mg/dL)	154 mg/dL
Creatinine (0.52-1.04 mg/dL)	0.48 mg/dL
Sodium (133-145 mEq/L)	140 mEq/L
Potassium (3.5-5.1 mEq/L)	3.5 mEq/L
Bicarbonate (22-30 mEq/L)	32 mEq/L
Calcium (8.4-10.5 mg/dL)	9.7 mg/dL
Anion gap (mEq/L)	27.00 mEq/L
Albumin (3.5-5.0 g/dL)	3.2 g/dL
Bilirubin total (0.2-1.3 mg/dL)	0.5 mg/dL
ALT (9-52 U/L)	38 U/L
AST (14-36 U/L)	24 U/L
Magnesium (1.6-2.3 mg/dL)	1.8 mg/dL
Lactate (0.70-2.10 mmol/L)	8.90 mmol/L
Troponin I (0.000-0.034 ng/mL)	0.031 ng/mL
pH, arterial (7.35-7.45)	7.45
pCO_2_, arterial (35.0-45.0 mmHg)	43.3 mmHg
pO_2_, arterial (80.0-110.0 mmHg)	150.0 mmHg
HCO_3_, arterial (22.0-26.0 mmol/L)	29.4 mmol/L
O_2_ saturation, arterial (96.0-97%)	99.0 %

## Discussion

It is well known that symptomatology of MG results from autoimmune destruction of nicotinic acetylcholine receptors located at the neuromuscular junction preventing nerve impulses from triggering muscle contractions. Although MG is a neuromuscular autoimmune disorder known for predominantly targeting skeletal muscles, there is a growing body of literature pointing towards cardiac involvement via anti-striational antibodies [3-5]. Acetylcholine receptor antibodies commonly seen in MG are specific to skeletal muscles and do not bind to heart muscle. However, anti-striational antibodies, including anti-titin, anti-RYR and anti-Kv1.4, do bind to cardiac muscle and have been reported to be present in 47% of the patients with MG [6]. Titin is a giant protein found in cardiac and skeletal sarcomere. RyR is a calcium release channel found in the sarcoplasmic reticulum with a role in regulating the excitation contraction coupling through calcium release. Kv 1.4 is a voltage-gated potassium channel. Both skeletal muscle and cardiac muscle are striated, and therefore these antibodies cross-react between skeletal and cardiac muscles, and are thought to trigger an inflammatory reaction and conduction abnormalities.

Association between MG and EKG abnormalities has also been investigated in several studies [7-9]. It was noted that ST depression, T-wave inversion, AV and right bundle branch block, as well as atrial fibrillation, have all been described. Anti-Kv1.4 antibodies have also been associated with EKG abnormalities including QT prolongation and T-wave inversion in 60% of the patients [10]. In the presence of myocarditis, anti-Kv 1.4 antibodies also have been associated with more significant EKG changes such as sick sinus syndrome, complete AV block and ventricular tachycardia.

In a study by Furlund Owe et al., EKG changes in 143 patients with MG were investigated and it was discovered that there was a shorter PR interval in patients with MG versus control group (156 ms vs 170 ms, p=0.047); however, the PR interval in MG group stayed within limits of normal (120-200 ms) [9]. In our patient, initial EKG demonstrated significantly shorted PR interval (96 ms) without delta wave or pre-excitation syndrome. MC with cardiac arrest and short PR interval, to our knowledge, was never described in the literature prior to this report. Our patient had a persistently short PR interval for the first days of his MICU stay. However, PR interval was normalized after the completion of multiple rounds of plasmapheresis (Figure 2). It is important to note that our patient remained on continuous cardiac monitoring during the length of his hospital stay and no significant arrhythmia was noted. It is thus possible to hypothesize that the short PR noted in our patient could be a manifestation of MC. The presence of a shortened PR interval may be an indicator of life-threatening arrhythmias leading to cardiac arrest.

Association between MG and myocarditis, arrhythmias and heart failure have all also been described in retrospective studies and case reports. Shivamurthy and Parker reported that they found 29 case reports and eight retrospective/prospective studies concerning cardiac involvement in MG [2]. A total of 48% of the patients were noted with MG and 97% of patients with MG and thymoma had presence of antibodies against cardiac muscle (anti-titin, anti-ryanodine and anti-Kv 1.4 antibodies) [4]. CT imaging on our patient did not reveal thymoma; however, as mentioned above, the absence of thymoma does not preclude presence of anti-striational antibodies. Testing for anti-striational antibodies is not available in the most institutions in the USA, including ours, and further studies are needed to establish value of those antibodies for cardiac screening in MG. The most common cardiac manifestation of MG found in the literature is myocarditis (37.5% MG patients with anti-striational antibodies) [2]. Severe myocarditis typically presents with chest pain, sign of cardiac failure, cardiogenic shock, and diagnosis based on elevation of troponin and plasma brain natriuretic peptide levels, cardiomegaly and decreased left ventricular ejection fraction. In our case, none of these mentioned was present which makes diagnosis of myocarditis unlikely.

## Conclusions

MC presenting as a cardiac arrest without other associated symptoms of MG is rare and diagnostically challenging, though the incidence of heart disease related to MC remains largely unknown. Clinicians should be aware of the possibility of cardiac manifestations of MG and be proactive in their approach to treating a cardiac MC since most respond to immunomodulatory therapy, as did our patient. Most published data consist of case reports or retrospective case-control studies that suggest an association of thymoma and anti-striational antibodies with cardiac involvement in MG. A short PR interval in MC can be an indicator of life-threatening arrhythmias. Prospective studies are needed to further establish recommendations for cardiac screening in MG.
